# Integrated Strategy of Network Pharmacological Prediction and Experimental Validation Elucidate Possible Mechanism of Bu-Yang Herbs in Treating Postmenopausal Osteoporosis via ESR1

**DOI:** 10.3389/fphar.2021.654714

**Published:** 2021-05-11

**Authors:** Hanting Xia, Jiangyuan Liu, Wenlong Yang, Min Liu, Yunfeng Luo, Zhijun Yang, Jingbo Xie, Huiliang Zeng, Rui Xu, Houfu Ling, Qinghe Zeng, Huihui Xu, Liang Fang, Hongyu Wang, Peijian Tong, Hongting Jin, Fengyun Yang

**Affiliations:** ^1^ The First Clinical College, Zhejiang Chinese Medical University, Hangzhou, China; ^2^ Institute of Orthopaedics and Traumatology, The First Affiliated Hospital of Zhejiang Chinese Medical University, Hangzhou, China; ^3^ Department of Orthopedics, Affiliated Hospital of Jiangxi University of Chinese Medicine, Nanchang, China; ^4^ Graduated School, Jiangxi University of Chinese Medicine, Nanchang, China; ^5^ Department of Orthopedics, People’s Hospital of Fengcheng City, Fengcheng, China; ^6^ Department of Orthopedics, Foshan Hospital of Traditional Chinese Medicine, Foshan, China; ^7^ Department of Orthopaedic Surgery, The First Affiliated Hospital of Zhejiang Chinese Medical University, Hangzhou, China

**Keywords:** network pharmacology, postmenopausal osteoporosis, traditional Chinese medicine, Bu-Yang, estrogen receptor

## Abstract

Postmenopausal osteoporosis (PMOP) is a type of bone metabolism disease-related to estrogen deficiency with an increasing incidence. Traditional Chinese (TCM) has always been used and showed effectiveness in treating PMOP. In the current study, Bu-Yang herbs were considered to be the most frequently used and efficient TCM herbs in PMOP treatment. However, chemical and pharmacological profiles were not elucidated. Network pharmacology was conducted on representative Bu-Yang herbs (Yin-Yang-Huo. Du-Zhong, Bu-Gu-Zhi, Tu-Si-Zi) to investigate the mechanism of Bu-Yang herbs on PMOP. Chemical compounds, potential targets, and disease related genes were available from the corresponding database. Results showed that Bu-Yang herbs could interact with ESR1 and estrogen signaling pathways. For further validation, the Bu-Yang decoction (BYD), formula consisted of the above-mentioned 4 Bu-Yang herbs was presented for experimental validation. *In vivo*, BYD significantly reversed ovariectomy (OVX)-induced osteoporosis progress in a dose-dependent manner by up-regulation of bone mineral density and amelioration of bone microarchitecture. *In vitro*, BYD dramatically improved the proliferation and mineral nodules formation of osteoblasts. Both *in vitro* and *in vivo* results illustrated that the phenotype change induced by BYD is correlated with up-regulated of ESR1 and activation of the β-catenin pathway. Meanwhile, inhibition of ESR1 by ICI182, 780 blocked the osteogenic phenotype and β-catenin pathway activation induced by BYD. In conclusion, the current study suggested that Bu-Yang herbs are the most useful TCM herbs in treating PMOP. Furthermore, the integrated strategy of network pharmacology prediction with experimental validation suggested that BYD exerted its anti-PMOP via ESR1 and the downstream mechanism might be activation of the β-catenin signaling pathway.

## Introduction

Postmenopausal osteoporosis (PMOP) is a systemic metabolic disease that frequently occurs in postmenopausal women, characterized by low bone mineral density (BMD) and damaged bone microarchitecture, resulting from unbalanced bone metabolism that leads to fragile bones and increased risk of fractures (Li et al., 2019a; Park et al., 2020a). A recent large-scale epidemiological study demonstrated the age-related prevalence of osteoporosis was 6.46 and 29.13% for men and women in the elderly (Zeng et al., 2019). PMOP is due to an imbalance in bone metabolism and attributed to excess bone resorption and insufficient bone formation due to estrogen deficiency (Kang et al., 2015; Black and Rosen, 2016). Estrogen deficiency is likewise viewed as a risk factor of PMOP (Kang et al., 2015). Estrogen is accepted to be a crucial role in regulating diverse physiological functions in the human body, including the reproductive system, energy, glucose and lipid metabolism, neurological system, and bone metabolism (Ryuk et al., 2017; Yang et al., 2019). A precipitous decline in postmenopausal women generates an estrogen-deficient internal environment that causes pathogenic alterations in bone homeostasis, leading to the occurrence of PMOP (Recine et al., 2019).

The biological effects of estrogen are transmitted by three estrogen receptors, the nuclear receptors estrogen receptor 1 (ESR1) and ESR2, and the transmembrane G protein-coupled estrogen receptor 1 (Hases et al., 2020). The major differences among these receptors happen to distribution, the phenotype corresponding to knock-out mice, and transcriptional activity (Lü et al., 2019). ESR1 is the major estrogen receptor subtype resident in bone tissue instead of ESR2 nor transmembrane protein-coupled estrogen receptor 1. ESR1-deletion eliminates the therapeutic effect of estrogen in the PMOP animal model (Streicher et al., 2017). Also, ESR1-deletion mice showed a phenotype of TNF-α regulation and spontaneous osteoporosis, suggesting that the ESR1 could be a major target in PMOP treatment (Raehtz et al., 2017). Thus, ESR1 could be the therapeutic target in treating PMOP. Estrogen replacement therapy (ERT) in PMOP patients increases the estrogen levels in the body and ameliorates PMOP-induced BMD loss and microarchitecture damage, but there is a controversial opinion over its safety for increasing the risk of endometrial cancer and breast cancer (Lan et al., 2017). Other conventional clinical therapies, including diphosphonate, parathyroid hormone, and calcium, have defects in efficacy or adverse effects too (Liu et al., 2020). Therefore, seeking new therapies in the view of natural products which have sufficient efficacy with minimal adverse effects in treating PMOP has attracted the interests of researchers.

Traditional Chinese medicine (TCM) has been recognized as a promising alternative therapy in treating PMOP for its satisfactory clinical efficacy and safety (Liu et al., 2017; N. Zhang et al., 2016). Different from one drug-one target concept of Western medicine, TCM emphasizes the concept of which the human body is just an organic whole (Zhou et al., 2020). With thousands of years of development, TCM has grown into a very intricate system and the ingredients of the TCM formula were many and complex (Han et al., 2018). The composition of traditional Chinese medicines is complex and diverse. Unlike Western medicine, which of the chemical compositions are simple and definite, the TCM formula is generally composed of complex matrixes and undefined active components which made it difficult to screen and analyze the exact bioactive components of the TCM formula (Bu et al., 2017).

Hopkins proposed the concept of “network pharmacology” and it’s becoming increasingly popular among researchers in recent years (Tang et al., 2020; Xu J. et al., 2019b). The characteristic of network pharmacology is following the holistic theory of TCM(S. He et al., 2019b). The basic opinion of network pharmacology is well suited for exploring the mechanisms of multi-components and multi-targets drugs, so it is an ideal approach in investigating and identifying the mechanism of the TCM formula (Guo et al., 2019; Huang et al., 2020; Liu et al., 2020; Zhang et al., 2019).

In the current study, we previously systematically reviewed all published literature reporting TCM treatment on PMOP and found that Yang-tonifying herbs (Bu-Yang) are most frequently used in TCM formula in treating PMOP, represented by Bu-Yang (Tonifying Yang) herbs, including Du-Zhong (DZ), Bu-Gu-Zhi (BGZ), Yin-Yang-Huo (YYH) and Tu-Si-Zi (TSZ). Detailed information was given in Table 1. Through the literature retrieval, Bu-Yang herbs were considered to be among the most effective TCM herbs for the treatment of PMOP. Understanding the mechanism of Bu-Yang herbs in treating PMOP would help us optimize the TCM formula strategy in treating PMOP. Hence, an integrated analytical platform was built based on network pharmacology, including target prediction, protein–protein interaction (PPI) network, topology analysis, KEGG analysis, and molecular docking prediction, was introduced to investigate the basic shared mechanism of Bu-Yang herbs in treating PMOP. ESR1 was expected to be the main target gene alongside the estrogen signaling pathway is the most enriched pathway of Bu-Yang herbs in treating PMOP.

**TABLE 1:** Detailed information of Yin-Yang-Huo, Du-Zhong, Bu-Gu-Zhi and Tu-Si-Zi.

Herbs	Latin name	Full taxonomic name	Place of origin	Weight (g)
Yin-Yang-Huo	*Epimedium brevicornum*	*Epimedium brevicornu* Maxim.	Gansu, China	10
Du-Zhong	*Cortex eucommiae*	*Eucommia ulmoides* Oliv.	Gansu, China	10
Bu-Gu-Zhi	*Malaytea scurfpea*	*Cullen corylifolium* (L.) Medik.	Yunnan, China	10
Tu-Si-Zi	*Semen cuseutae*	*Cuscuta chinensis* Lam.	Hebei, China	12

Bu-Yang decoction (BYD), composed of YYH, DZ, BGZ, and TSZ, was introduced for experimental validation. We investigated the effects of BYD on treating PMOP via ESR1 *in vivo* and *in vitro*. Results validated that the ESR1 is the primary target of BYD on treating PMOP, inhibiting ESR1 reversed the therapeutic effects of BYD. Furthermore, Wnt/β-catenin was found to be up-regulated in osteoblasts in the presence of BYD, inhibiting ESR1 also blocked the activation of Wnt/β-catenin. This could be the down-stream mechanism of BYD treatment.

**FIGURE 1:**
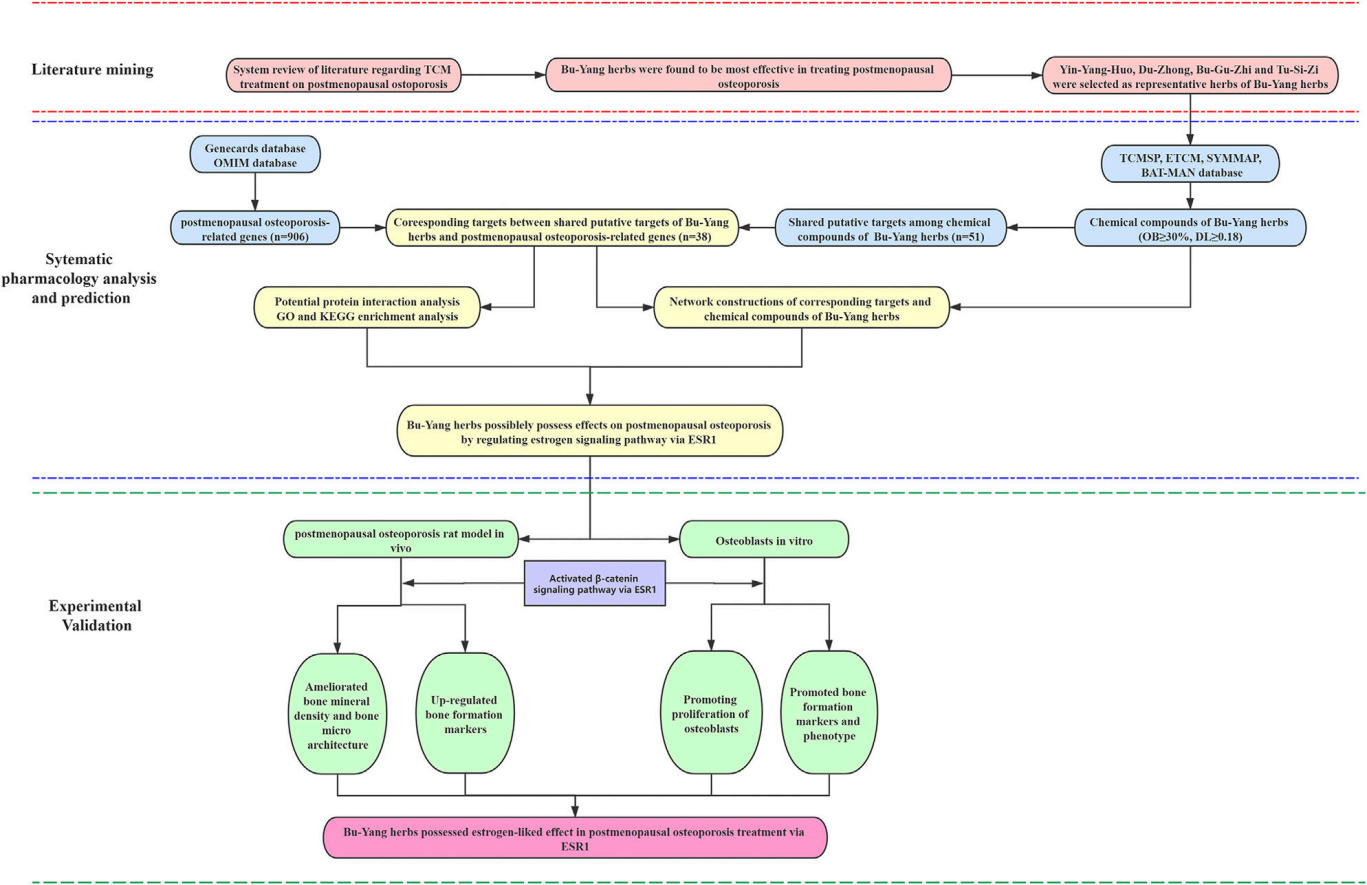
A combined strategy of literature mining, systematic pharmacology, and experimental validation in investing TCM treatment on PMOP.

In summary, systematically literature reviewing combines with network pharmacology of Bu-Yang herbs on PMOP to predict the active compounds and potential target genes and pathways. Besides, BYD *in vitro* and Vivo experimental validation was conducted to reveal the underlying mechanism of BYD on PMOP, as previously predicted by network pharmacology. The detailed strategy of this study was shown in Figure 1.

## Methods

### Literature Mining

To determine the potential effective TCM herbs along with the TCM categories of these herbs in the treatment of PMOP, a review of the literature was performed with PubMed database, CNKI database (in Chinese), and VIP database (in Chinese). The search term was set to be as follow: Osteoporosis [ti/ab] AND (bone mineral density [ti/ab]) AND (traditional Chinese medicine [ti/ab]). 2 of the authors, Hanting Xia and Wenlong Yang were designated to be the reviewer of the collected literature. Reviewing was conducted dependently with the following criterion: a clinical or experimental study reported the TCM, regardless of formula or single herb, with an effect of ameliorating the bone mineral density (BMD) in OP animal model or OP human, respectively. The exclusion criterion included combined with other therapies, BMD change was not reported or poor reliability of the study.

All counted herbs were attributed to corresponding TCM property according to TCM theory. According to the statistical result, the most attention-attracted category of TCM herbs in treating OP, which is Bu-Yang (Tonifying Yang) herbs, including Du-Zhong (DZ), Bu-Gu-Zhi (BGZ), Yin-Yang-Huo (YYH), and Tu-Si-Zi (TSZ), were adopted for further analysis. Detailed information was given in Table 1.

### Systematic Pharmacology Data Preparation

The chemical components of TCM herbs of YYH, DZ, BGZ, and TSZ were conducted by a combined searching and collection in the online database including TCMSP (Ru et al., 2014), ETCM (Xu et al., 2019a), SYMMAP (Wu et al., 2019) and BAT-MAN (Liu et al., 2016). The search was done on September 20, 2018. Absorption, distribution, metabolism, and excretion (ADME) parameters were introduced to screen the potential drugs of all collected chemical compounds of YYH, DZ, BGZ, and TSZ. The screening criterion was set to be Oral Bioavailability (OB) ≥ 30% and Drug Likeness ≥ 0.18 and applied to the database. After screening, the resultant chemical compounds were defined to be putative active compounds of corresponding herbs, and the structure of these chemical compounds was collected on the PubChem database. Meanwhile, qualified putative targets of the potential active compounds were collected on these network databases.

### Known Therapeutic Targets of Postmenopausal Osteoporosis

Known therapeutic targets of PMOP were collected by a combined search in the online database including Genecards (Safran et al., 2003) and OMIM (Hamosh et al., 2005). Searching keywords were set to be “postmenopausal osteoporosis.” Resulted related target genes were demonstrated in table on the webpage and the download the page. Additionally, Drugbank (Wishart et al., 2018) was also searched for FDA-approved drugs in treating PMOP and the targets of these FDA drugs were also collected in this study. Briefly, the search term was set to be “postmenopausal osteoporosis,” enter the disease “postmenopausal osteoporosis” and shift to the “drug and target” label page, collect all targets. Resulted target genes from Genescards, OMIM, and Drugbank database were collected in the form of abbreviation and the duplications were removed by the excel software.

### Visualized Network Construction and Analysis

Interesting genes, like shared genes between PMOP and the herbs or the known therapeutic targets of PMOP along, were analyzed with STRING (Szklarczyk et al., 2019) online tool to obtain the Protein-protein interaction (PPI) information. Alternatively, DAVID (Huang et al., 2009) tool was used to conduct the Gene Ontology analysis and KEGG pathway analysis. The interaction between herbs, compounds, targets, and enriched signaling pathways was used as modulates to construct a network with Cytoscape software.

### Molecular Docking Prediction

Refer to previous studies (Tao et al., 2020; Xia et al., 2020), use AutoDock Tools 1.5.6 software to remove hydrone from ESR1, separate ligands, and receptors, add non-polar hydrogen, calculate Gasteiger charge, and save as pdbqt file. Download the small molecule 3D coordinate file from PubChem, including icarrin, (+)-Pinoresinol, beta-sitosterol, kaempferol, medioresinol, and quercetin, check the spatial structure in pymol for errors, and convert to PDB format using open babel. Load the structural parts into the AutoDock Tools 1.5.6 program, add atomic charges, assign atomic types, all flexible bonds are rotatable by default. Save in pdbqt format as docking ligand. Using ESR1 as the acceptor and the Chinese medicine candidate as the ligand, determine the active site for molecular docking according to the coordinates of the ligand in the target protein complex, set the Gridbox coordinates and size according to the ESR1 active pocket, perform molecular docking using AutoDock Vina, and analyze and process the docking results. The lowest binding energy was used as the result of the target protein and ligand docking. The score ranges from −0.0 to −10.0. Generally, the absolute value of the score is greater than 5, the compound would be viewed as has a possible interaction with ESR1. The closer the absolute value of the score is to 10, the stronger potential interaction with ESR1 the compound has.

### Bu-Yang Decoction Preparation

For experimental validation of effects and mechanisms of Bu-Yang herbs. YYH, DZ, BGZ, and TSZ were combined as a formula and named as Bu-Yang decoction (BYD). Referred to 2015 Chinese Pharmacopoeia, the maximum dosage was adopted, 10 g YYH, 10 g DZ, 10 g BGZ, 12 g TSZ. All herbs were purchased from the Affiliated Hospital of Jiangxi University of traditional Chinese medicine. All crude herbs were soaked in four times a volume of water and boiled for 30 min followed by filtration twice. The resultant decoction was concentrated and dried to be decoction powder. Dry decoction 8.4 g were collected per 42 g mixed crude herbs. Extraction was diluted and stored at 4°C. Furthermore, the BYD solution was filtered with 0.22 μm filter (Millipore, Billerica, MA, United States) for subsequent *in vivo* experiments. Detailed information of used herbs was shown in Table 1.

### Chemical Components and Quality Control of Bu-Yang Decoction

To determine the main chemical components of BYD, LC-MS analysis was conducted. 200 mg dry Bu-Yang decoction was added with 1 ml methanol, whirled for 10 min, centrifuged at 4°C for 10 min. Centrifugal force was set to be 20,000 xg. The supernatant was collected and filtered with a 0.22 µm filter membrane. The resultant sample was subjected to chromatographic analysis on an Ultimate 3000 RS system (Thermo Fisher Scientific, MA, United States) equipped with a Thermo Hypersil GOLD column (φ 2.1 × 100 mm, 1.9 µm) and the MS spectra were acquired by a Q Executive high-resolution mass spectrometer (Thermo Fisher Scientific, MA, Utates States).

The mobile phases were (A) 0.1% formic acid in water (B) and 0.1% formic acid in acetonitrile, and the gradient elution program was (time/B%): 0–1 min, 2%; 1–5 min, 2–20%; 5–10 min, 20–50%; 10–15 min, 50–80%; 15–20 min, 80–95%; 20–25 min, 95%; 26–30 min, 2%. The chromatographic analysis was performed at 35°C with a flow rate of 0.3 ml/min and an injection volume of 15 µL. The mass spectrometer parameters were as follows: spray voltage was set at 3.8 kV at positive mode. The capillary temperature was set at 300°C. Argon was used as the collision gas. Nitrogen was used as sheath gas and aux gas. Aux gas heater temperature was set to be 350°C. Resultant data were analyzed by CD 2.1 software (Thermo Fisher Scientific, MA, United States) and then compared with online databases.

### Cell Isolation and Culture

Osteoblasts were obtained from the cranium of newborn rats housed in the Laboratory Animal Science and Technology Center of Jiangxi University of TCM (Nanchang City, Jiangxi Province, China). Specimens were cut into pieces and then digested with 0.25% trypsin-EDTA solution (Solarbio, Beijing, China) for 1 h followed by 0.1% collagenase I (Solarbio, Beijing, China) for 4 h to obtain osteoblasts. Osteoblasts were seeded in 25 cm^2^ culture flasks and cultured with DMEM (Solarbio, Beijing, China) containing 10% FBS (Gibco, NY, United States) and antibiotics (100 U/ml penicillin, 0.1 mg/ml streptomycin) in 5% CO_2_ at 37°C conditions.

Osteoblast were analyzed by immunofluorescence using the β‐catenin primary antibody followed by respective secondary IgG (H+L) antibodies. Visualizing images were conducted by Leica Confocal Microscope.

Osteoblasts were cultured in 6‐well plated culture plate for 24 h at 37°C. After incubation, cells were washed, and followed by culture medium for induction of osteogenic differentiation for 21 days. The cells were fixed after air‐drying and left for 30 min at −20°C. The osteoblasts were stained with 221 alizarin red S at room temperature. After washed, osteoblasts were observed by Leica Confocal 222 Microscope.

### MTT Assay

Osteoblast’s proliferation was detected by MTT assay. An initial density of 0.5 × 104/well osteoblasts was applied to 96-well plates, respectively. After 12 h of serum-starved incubation, the various concentration of BYD was added to the medium (50, 100, 200, 400 μg/ml). 24 and 48 h treatments later, 20 μL MTT solution (Solarbio, Beijing, China) at a concentration of 5 mg/ml was mixed with the medium. Removed the supernatant and 150 μL DMSO solution (Solarbio, Beijing, China) was added. Optical density was measured at 570 nm with a multimode reader (Spark 10M, TECAN, Switzerland).

### Animal Study

60 female Sprawl-Dawley rats (200 ± 20 g) were purchased from the Laboratory Animal Science and Technology Center of Jiangxi University of Traditional Chinese Medicine (Nanchang City, Jiangxi Province, China). All rats were free to access to diet and water. All animal care and protocols were approved by the Committee of Management and Use of Laboratory Animals of Jiangxi University of Traditional Chinese Medicine (Nanchang City, Jiangxi Province, China). All animal experiments complied with the Guide for the National Institutes of Health guide for the care and use of laboratory animals.

PMOP rat model established by operatively ovariectomized refer to the previous study (Streicher et al., 2017). Briefly, all rats except the sham group, were anesthetized by the intraperitoneal injection of pentobarbital and skin was prepared. Rats were fixed in the supine position for surgery. After general anesthesia, the operation area was disinfected, and the towels were spread. A longitudinal incision was made, and the skin and subcutaneous tissue were incised. The abdomen was opened, and the uterus was located and pulled out of the abdominal cavity. Cut off the ovary. The same procedure was performed on the opposite side to excise both ovaries. As for the Sham group, the ovaries of rats were reserved and only similar weight fat to the ovary was excised. All rats were randomly divided into 5 groups equally: 1) sham-operated, 2) bilaterally ovariectomies (OVX), 3) OVX rats treated with 0.75 g/kg/d BYD, 4) OVX rats treated with 1.5 g/kg/d BYD, 5) OVX rats treated with 3.0 g/kg/d BYD and 6) OVX rats treated with 487.5 μg/kg/d β-estradiol (Macklin, Shanghai, China).

Blood samples were collected for enzyme-linked immunosorbent assay (ELISA). All rats were sacrificed after 12 weeks of treatment. Femur tissue was collected for further analyses.

### Western Blot

Total protein samples were extracted using a protein extraction kit following the manufacturer’s instructions. Protein samples were separated by sulfate polyacrylamide gel electrophoresis and then blotted onto polyvinylidene fluoride membranes. The proteins on the blot were assessed using primary antibodies against ESR1 (Abcam, Cambridge, United Kingdom), RUNX2 (Abcam, Cambridge, United Kingdom), β-catenin (Proteintech, Chicago, United States), cyclinD1 (Abcam, Cambridge, United Kingdom), and β-actin (Abcam, Cambridge, United Kingdom) followed by secondary antibodies. Blots were visualized using the electrochemiluminescence (Thermo Scientific, NY, United States) method. The density of protein bands was quantified with Image Lab software (Bio-Rad, CA, United States).

### RT-PCR

Total cellular RNA was isolated using TRIzol reagent (Invitrogen, Carlsbad, USA) according to the manufacture’s instruction. Complementary DNA (cDNA) was reverse‐transcribed with PrimeScript‐RT reagent kit (TaKaRa Biotechnology Co, Ltd., Japan). The mRNA levels of ESR1, β‐catenin, CyclinD1, RUNX‐2 were detected by quantitative real-time PCR with SYBR Premix Ex Taq kit (TaKaRa Biotechnology Co, Ltd., Japan). Primer sequences used are shown in Supplementary Table S1 and the specificity of sequences was verified using the BLAST algorithm of National Center for Biotechnology Information. Data were normalized and analyzed by 2(−ΔΔCT) method.

### Micro-CT Scan and Bone Mineral Density Analysis

Micro-CT scan (Skyscan 1176, Bruker micro CT N.V, Kontich, Belgium) and 3-dimension remodeling was applied for radiographic observation. The right distal femur was harvested and cut to fit the appropriate size for micro-CT scan. Bone volume/Trabecular Volume (BV/TV), Trabecular separation (Tb. Sp), number of trabecular (Tb. N) and bone mineral density (BMD) were calculated by analysis software version 1.1.1.

### ELISA Analysis

*In vitro*, 6 duplications of osteoblasts were seeded at 24-well plates with or without 200 mg/ml BYD for 24 and 48 h in 37°C, respectively. After treatment, washed twice with PBS. Subsequently, osteoblasts were lyzed with 0.2% Triton X-100 and centrifuged. The supernatant was collected for ALP activity. Optical density at respective nm was measured with a multimode reader.

*In vivo*, samples of rat plasma were collected. The supernatant liquid was collected after centrifugation. These samples were used for ELISA assay for OPG, ALP, and PINP according to the manufacturer’s instructions. Briefly, 5 gradient concentrations were applied with 10 duplications. Rat plasma was added to the 96-well plate and followed by incubation at 37°C. Washed with distilled water, an enzyme reagent was added followed by a chromogenic agent. Optical density was measured at 450 nm with a multimode reader (Spark 10M, TECAN, Switzerland).

### Statistical methods

Data were presented as mean ± Stand Deviation and analyzed using GraphPad Prism 8 software. One-way analysis of variance (ANOVA) was introduced. *p* value < 0.05 was considered statistically significant and exact *P* value was shown in respective figures.

## Results

### Bu-Yang Herbs Were Most Frequently Used TCM Herbs in Postmenopausal Osteoporosis Treatment

An amount of 1,559 records were collected, 1,353 in Chinese, 206 in English. All records were scrutinized by 2 independent reviewers. Inclusion and exclusion criteria were shown in Supplementary Figure S1A. 483 records were included for further analysis.

According to the 483 records, 112 TCM herbs attributed to 17 different categories were reported to be effective in treating PMOP. As shown in Supplementary Figure S1B, Bu-Yang herbs were reported 601 times, which is the most frequently used TCM herbs category in treating PMOP, including YYH (194 times), Du-Zhong (96 times), Bu-Gu-Zhi (74 times), Lu-Jiao-Jiao (Colla Cornus Cervi, 59 times), Tu-Si-Zi (51 times) and others. The rest kinds of TCM categories were reported significantly less than Bu-Yang herbs, including Bu-Xue (Nourishing Blood) herbs, Huo-Xue-Hua-Yu (Invigorating blood circulation and eliminating stasis) herbs, Bu-Qi (Tonifying Qi) herbs, Bu-Yin (Nourishing Yin), Bu-Gan-Shen (Nourishing Liver and Kidney) herbs were reported 286 times, 274 times, 214 times, 105 times, 73 times, respectively. Thus, YYH, DZ, BGZ, and TSZ were selected to be representative Bu-Yang herbs for further network pharmacology analysis. Notably, LJJ was excluded for being animal-derived TCM medicine instead of herb and no sufficient data of LJJ was searched in the online TCM herb database.

### ESR1 and Estrogen Pathway Were Predicted to be the Mechanism of Bu-Yang Herbs in Treating Postmenopausal Osteoporosis

According to the criteria of DL ≥ 0.18 and OB ≥ 30%, the online database showed YYH, DZ, BGZ, and TSZ had 70, 80, 20, and 11 qualified chemical compounds, respectively. 1,180 genes were predicted to be the targets of these compounds, which belonged to the relative herb. 51 genes were found to be the shared targets of all 4 herbs, as shown in Supplementary Figures S2A,B. These 51 genes were viewed as the shared fundamental targets of YYH, DZ, BGZ, and TSZ.

Furthermore, 906 genes were collected as PMOP disease-related genes by the online database. To investigate the potential therapeutic target genes of YYH, DZ, BGZ, and TSZ in treating PMOP, Venn analysis was conducted and found that 38 genes were viewed as the cross-talking genes between Bu-Yang herbs and PMOP, as shown in Supplementary Figure S2C. Refer to Li’s study (Li et al., 2007), hub genes were identified as the nodes with a degree equal or greater than twice the median of all nodes. Results showed that ESR1, ALB, TP53, and AKT1 were hub genes in the PPI network, indicated that these 4 genes might play a pivotal role in YYH, DZ, BGZ, and TSZ treatment on PMOP, as shown in Supplementary Figure S2D. Meanwhile, according to the compounds-target network, as shown in Supplementary Figures S2A,E, only 20 genes interacted with more than 10 different chemical compounds, including ESR1, PTGS2, and others, which indicates that these 20 genes might possess a fundamental effect, detailed information seen in Supplementary Table S2.

**FIGURE 2:**
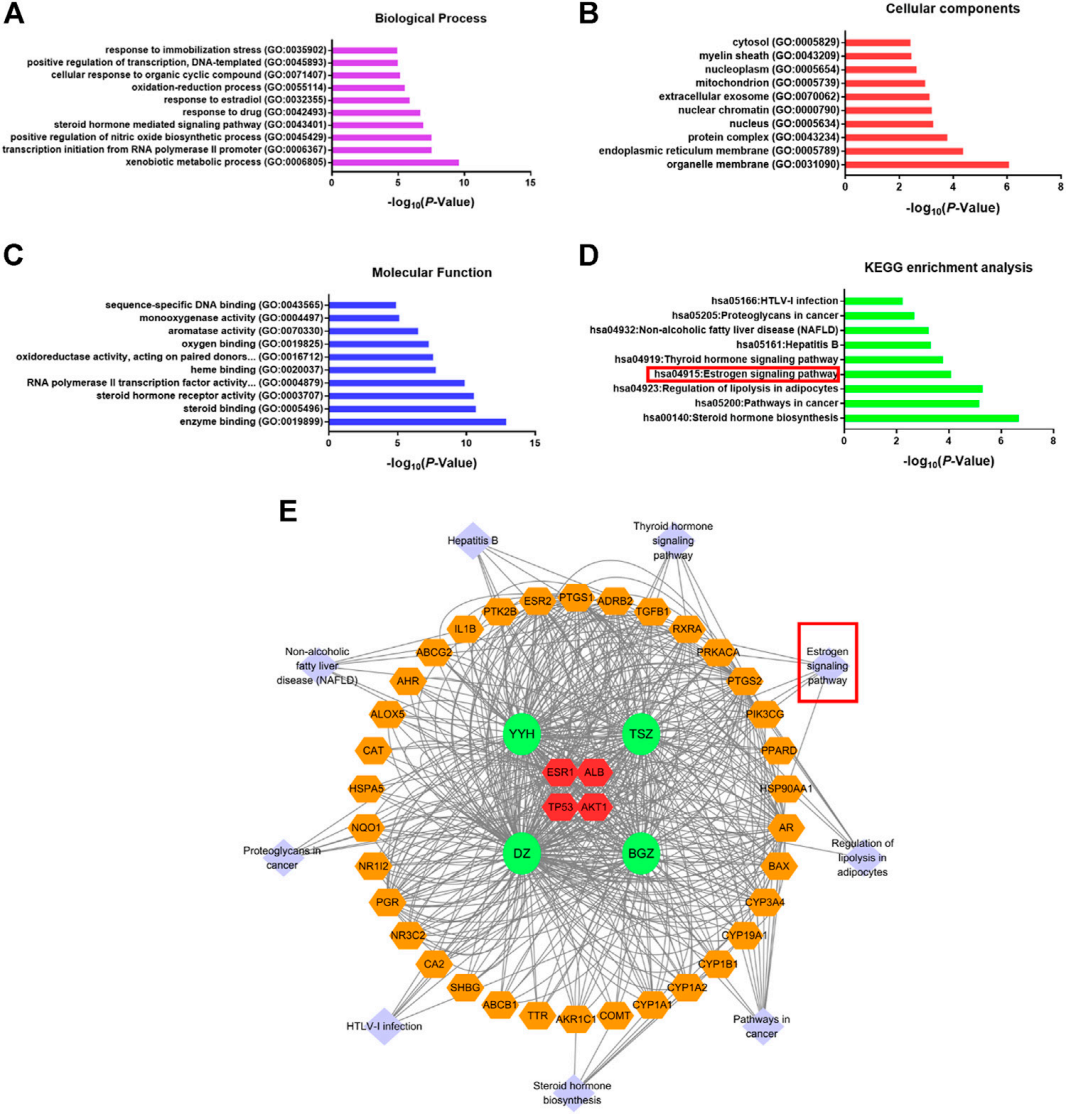
Gene ontology and KEGG enrichment analysis. Gene ontology analysis of 38 cross-talking genes was displayed in 3 modules, including **(A)** biological process, **(B)** cellular components, and **(C)** molecular function. **(D)** KEGG signaling pathway enrichment analysis. **(E)** Pathway-herb-cross-talking genes network. The orange hexagon represents the same as in Figure 3. The light purple diamond represents the pathways enriched by 38 cross-talking targets. The green circle represents the herb.

Gene ontology (GO) and KEGG pathway enrichment analysis of 38 cross-talking targets were conducted by the DAVID database (Huang et al., 2009), as shown in Figure 2. Notably, the estrogen signaling pathway was found to be one of the most relevant signaling pathways of Bu-Yang herbs treatment on PMOP. In line with our previous finding indicated that ESR1 was the crucial gene. Considering the fundamental role of estrogen in PMOP pathogenesis, the current study inferred that ESR1 and estrogen signaling pathway was the mechanism of Bu-Yang herbs on PMOP.

### LC-MS Analysis and Quality Control of Bu-Yang Decoction

For further experimental validation, representative Bu-Yang herbs, YYH, DZ, BGZ, and TSZ, were combined as Bu-Yang decoctions (BYD).

In the current study, LC-MS was conducted to detect the content of icariin, pinoresinol, quercetin, and kaempferol in BYD. According to the previous network pharmacology analysis, these 4 chemical compounds were found to be shared by at least 2 herbs of BYD, thus the content of them was considered as the standard to evaluate the quality of BYD in this study. As shown in Supplementary Figure S3A, 6 independent batches of BYD were analyzed by LC-MS. Overlapping chromatograms showed in Supplementary Figure S3 illustrated that the stability of BYD was confirmed. Detailed information about representative compounds of BYD was shown in Supplementary Table S3 **.**

### BYD Reversed OVX-Induced BMD Loss and Micro Architecture Damage on Postmenopausal Osteoporosis Rats *In Vivo*

**FIGURE 3:**
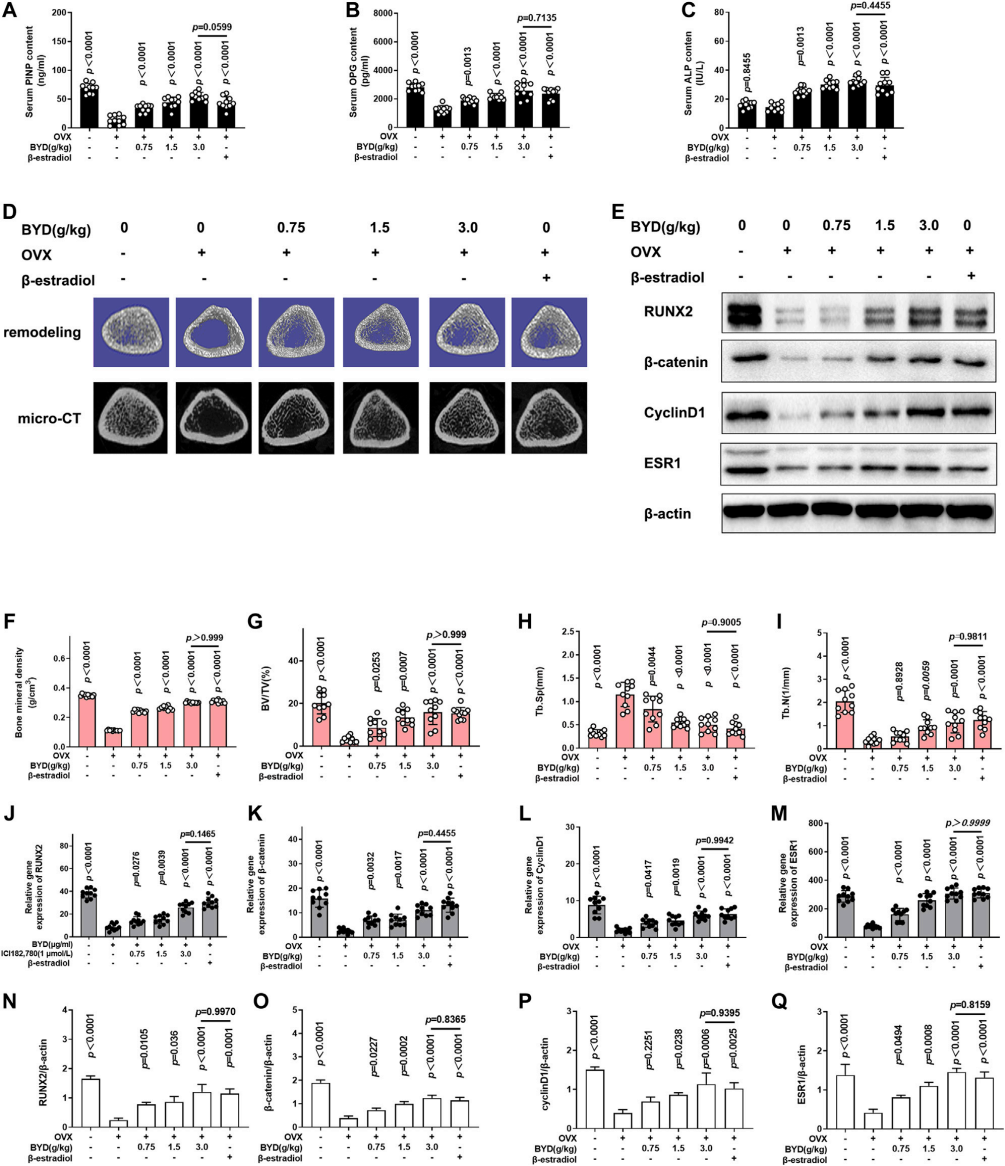
BYD promoted bone mineral density and microarchitecture of bone in OVX-rats in a dose-dependent manner via ESR1. Bone metabolism index of serum **(A)** PINP, **(B)** OPG, and **(C)** ALP, detected by ELISA (D) Representative images of micro-CT scanning and 3-dimensional remodeling of the distal tibia. **(E)** Representative images of western blots of RUNX2, β-catenin, CyclinD1, and ESR1. Quantitative results of bone morphometry, including **(F)** Bone mineral density, **(G)** BV/TV, **(H)** Tb.Sp, and **(I)** Tb.N. Quantitative analysis results of mRNA expressions of **(J)** RUNX2, **(K)** β-catenin, **(L)** CyclinD1 and **(M)** ESR1, Quantitative analysis results of protein expressions of **(N)** RUNX2, **(O)** β-catenin, **(P)** CyclinD1 and **(Q)** ESR1. THE exact *p*-value can be found in the corresponding histogram.

Micro-CT scanning with 3-dimensional remodeling and serum bone metabolism index analysis was applied on OVX-induced PMOP rats to validated the anti-PMOP effect of BYD *in vivo*. As shown in Figures 3A–C, ELISA was conducted to evaluate whether BYD increased the serum osteogenic markers, the results revealed that serum PINP and OPG were significantly down-regulated in OVX-induced PMOP rats (*p* < 0.0001), no significant change of serum ALP was found in OVX-induced PMOP rats (*p* = 0.8455). Intragastric administration of BYD for 12 weeks reversed the trend of serum PINP and OPG, compared to the OVX-induced PMOP group in a dose-dependent manner. Also, BYD up-regulated the serum ALP in a dose-dependent manner. The high dosage of BYD at 3.0 g/kg/day showed the most effective result. Meanwhile, β-estradiol treatment promoted serum PINP, OPG, and ALP, compared to the OVX-induced PMOP group (*p* < 0.0001). No significant differences were found between the β-estradiol-treated group and 3.0 g/kg/day BYD group (*p* = 0.0599 for serum PINP, *p* = 0.7135 for serum OPG, and *p* = 0.455 for serum ALP, respectively).

Figure 3D showed the representative images of micro-CT scanning and 3-dimensional remodeling of the distal femur and the quantitative analysis results were shown in Figure 3D. Figure 3D demonstrated that there are more trabecular in the sham group, and the resident trabecular were more compact and thick. In OVX-induced PMOP rats, the micro architecture of bone was damaged, the numbers of trabecular were reduced and they are sparse. Both BYD treatment and β-estradiol treatment reversed the trend. The quantitative analysis results confirmed the scanning results, revealed that BMD, trabecular BV/TV, and Tb. N was significantly decreased in OVX-induced PMOP rats compared to the sham group (*p* < 0.0001). BYD treatment reversed the trend in a dose-dependent manner in a dose-dependent manner, shown in Figures 3F–I. Inconsistent with the serum ELISA analysis, no significant differences were found between the β-estradiol group and 3.0 g/kg/d BYD (*p* > 0.999 for BMD, *p* > 0.999 for BV/TV, *p* = 0.9005 for Tb. Sp and *p* = 0.9811, respectively). Results showed that BYD protected bone mineral density and bone micro architecture from OVX-induced estrogen-deficiency-related osteoporosis and the high dosage at 3.0 g/kg/d BYD showed the anti-PMOP effect no worse than β-estradiol.

### BYD Promoted the Osteoblasts Proliferation and Osteoblastic Function *In Vitro*

**FIGURE 4:**
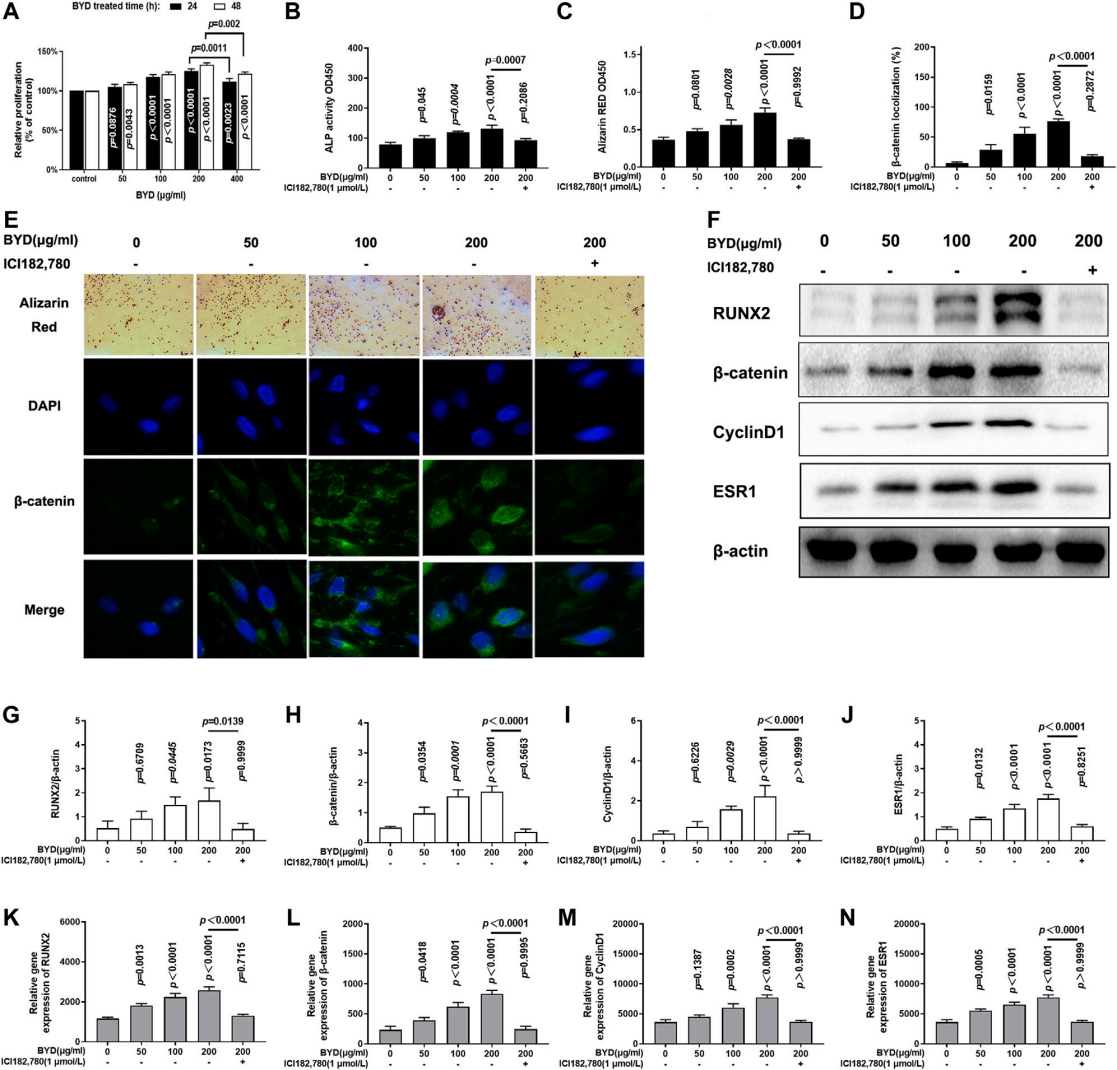
BYD activated Wnt/β-catenin signaling pathway to the promoted osteoblastic formation *in vitro* via ESR1. **(A)** Relative proliferation of various concentration of BYD (50, 100, 200, 400 μg/ml). **(B)** Alkaline phosphatase (ALP) activity. **(C)** Result of Alizarin Red staining. **(D)** β-catenin localization analysis result. **(E)** Representative images of alizarin red staining and immunofluorescence staining of β-catenin. **(F)** Representative images of western blots of RUNX2, β-catenin, cylindD1, and ESR1. Quantitative analysis results of protein expressions analysis of **(G)** RUNX2, **(H)** β-catenin, **(I)** CyclinD1, and **(J)** ESR1. Quantitative analysis results of mRNA expression analysis of **(K)** RUNX2, **(L)** β-catenin, **(M)** CyclinD1, and **(N)** ESR1. THE exact *p*-value can be found in the corresponding histogram.

The effect of BYD on osteoblasts proliferation was assessed at various concentrations of BYD (50, 100, 200, and 400 μg/ml) treated osteoblasts for 24 h or 48 h, as shown in Figure 4A. BYD promoted proliferation of osteoblasts in a dose-dependent manner in the range of 50, 100 and 200 μg/ml BYD treatment for both 24 h (*p* = 0.0876, *p* < 0.0001, *p* < 0.0001, for 50, 100, 200 μg/ml BYD, respectively) and 48 h (*p* = 0.043, *p* < 0.0001, *p* < 0.0001, for 50, 100, 200 μg/ml BYD, respectively). Mild cytotoxicity effect was found in osteoblasts incubated with 400 μg/ml BYD for both 24 h (*p* = 0.0011, compared with 200 μg/ml BYD) and 48 h (*p* = 0.002, compared with 200 μg/ml BYD). Additionally, the proliferation of BYD-treated osteoblasts for 48 is more prominent compared to the relative dosage group for 24 h. Thus, various concentrations of 50, 100, and 200 μg/ml BYD treated for 48 h were applied for further analysis *in vitro*.

ELISA analysis was conducted to determine whether BYD promoted the expressions of osteogenesis biomarkers. Figures 4B,C demonstrated that BYD up-regulated the content of ALP in a dose-dependent manner (*p* = 0.045, *p* = 0.0004, *p* < 0.0001, for 50, 100, 200 μg/ml BYD, respectively). Furthermore, Alizarin Red staining was done on osteoblasts to evaluate the effects of BYD on promoting bone formation. As shown in Figures 4C,E, BYD-treated osteoblasts formed more mineralization nodules in a dose-dependent manner compared to control (*p* = 0.0801, *p* = 0.0028, *p* < 0.0001, for 50, 100, 200 μg/ml BYD, respectively). Thus, results showed that BYD could promote the proliferation and osteoblastic function of osteoblasts *in vitro*.

### BYD Activated β-Catenin Signaling Pathway via ESR1

As a previously mentioned prediction, network analysis predicted ESR1 is the main target of BYD in treating PMOP. RUNX2 is a biomarker for osteogenesis. β-catenin and cyclinD1 are the downstream targets of the β-catenin signaling pathway, which is involved in bone genesis. Western Blot and RT-PCR were applied to determine whether BYD exerted its anti-PMOP effect via ESR1 and the possible downstream targeting pathways. As shown in Figures 3F–I, RT-PCR results revealed that the protein expressions of ESR1, RUN2, β-catenin, cyclinD1, and ESR1 were significantly decreased in OVX-induced PMOP rats compared to the sham group (*p* < 0.0001). The treatment of BYD reversed the trend in a dose-dependent manner. The high dosage of 3.0 g/kg/d BYD possessed the most prominent effect and was similar to β-estradiol treatment. The same trend was also confirmed in protein level by western blots, as shown in Figures 3E,N–Q. The results *in vivo* suggested that BYD-induced up-regulation of ESR1 might lead to the activation of the β-catenin signaling pathway. This could be the possible downstream mechanism of BYD in treating PMOP. For further validation, ICI182,780, an inhibitor of estrogen receptor was introduced to validate the effect of BYD on ESR1 *in vitro*.

As shown in Figures 4B,C,E, the addition of ICI182,780 significantly reversed the BYD-induced up-regulation of osteogenic activities, including ALP and Alizarin red staining, compared to 200 μg/ml BYD treated alone (*p* = 0,007, for ALP and *p* < 0.0001 Alizarin red staining). Additionally, no differences were found between ICI182, 780 co-treatment, and control (*p* = 0.2086 for ALP and *p* = 0.9992 for Alizarin red staining). Results showed that BYD promoted osteogenic function relied on ESR1, inhibition of ESR1 blocked the up-regulation effect of BYD on osteoblasts.

Moreover, mRNA and protein expressions of RUNX2, β-catenin, cylicinD1, and ESR1 were also detected *in vitro*. Figures 4G–J demonstrated that the protein expressions of RUNX2, β-catenin, cyclinD1, and ESR1 were significantly up-regulated in BYD treated osteoblasts in a dose-dependent manner and ICI182,780 co-treatment reversed the trend compared to the 200 μg/ml BYD (*p* = 0.0139, *p* < 0.0001, *p* < 0.0001 and *p* < 0.0001 for RUNX2, β-catenin, cyclinD1, and ESR1, respectively). ICI182,780 co-treated osteoblasts showed no difference compared to the control group (*p* = 0.9999, *p* = 0.5663, *p* = 3,630, and *p* > 0.9999 for RUNX2, β-catenin, cyclinD1, and ESR1, respectively). Figures4K–N demonstrated that the mRNA expression trends of RUNX2, β-catenin, cyclinD1, and ESR1, were similar to the results of protein expressions mentioned above: BYD up-regulated the mRNA expressions of these genes and were blocked by ICI182,780 co-treatment. Meanwhile, immunofluorescence was conducted to verify the nuclear translocation of β-catenin, which is the main feature of the activation of the β-catenin signaling pathway, as shown in Figures 4D,E. β-catenin was significantly accumulated and translocated to the nucleus of osteoblasts after BYD treatment (*p* = 0.0159 for 50 μg/ml BYD, *p* < 0.0001 for 100 and 200 μg/ml BYD), and co-treated with ICI182, 780 blocked the trend induced by BYD (*p* < 0.0001). Results demonstrated that BYD activated β-catenin signaling pathway and RUNX2 via ESR1, inhibition of ESR1 blocked the β-catenin signaling pathway, and reversed the osteogenesis phenotypes in BYD treated osteoblasts.

## Discussion

Traditional Chinese medicine has long been used for many years to treat PMOP in China and has been shown to have both anabolic and anti-catabolic effects in treating PMOP by alleviating unbalanced bone formation and resorption (He et al., 2019a). TCM formula is usually composed of multiple compounds, therefore TCM formula usually possessed complicated pharmacological effects via multiple targets and pathways (Zhou et al., 2014), which might contribute to alleviating the progressing of PMOP. However, the “multi-compounds-multi targets-multi mechanisms” characteristic of the TCM formula impeded the elucidation of active ingredients and the underlying mechanisms of TCM in treating PMOP. Systematic biology, represented network pharmacology, provided a different perspective to explore the exact pharmacological mechanisms by which TCM alleviated PMOP.

The key question to ask is to determine what kinds of TCM herbs were most efficient and frequently used in PMOP treatment. In the current study, a systematic searching and screening strategy were conducted among online databases, including PubMed in English and CNKI in Chinese, for literature reviewing to determine the most frequently reported kinds of TCM herbs to be effective in treating PMOP kinds of TCM herbs. Results revealed that Bu-Yang herbs were most frequently utilized in PMOP treatment among all TCM herbs, represented by YYH, DZ, BGZ, and TSZ. Subsequently, network pharmacology analysis of YYH, DZ, BGZ, and TSZ was done to determine the possible functional chemical compounds and potential targets. Considering that certain chemical compounds lacking appropriate pharmacokinetics properties cannot bind effectively to the target or produce pharmacological effects, refer to the previous study (Guo et al., 2019; Liu et al., 2020; Zhang et al., 2019), OB ≥ 30% and DL ≥ 0.18 were viewed as pharmacokinetically effective to be absorbed and utilized by oral administration in this study.

Based on the compound-target network analysis, chemical compounds commonly contained by more than one herb might account for the basic shared therapeutic effects of Bu-Yang herbs on PMOP. Several chemical compounds were identified, including quercetin, kaempferol, beta-sitosterol, pinoresinol, medioresinol. Quercetin, a natural flavonoid abundantly found in fruits and vegetables, was found to inhibit RANKL-induced osteogenesis, osclastgenteoblasts apoptosis, oxidative stress, and inflammatory response while restoring the balance between bone anabolism and catabolism via multiple pathways including β-catenin, NF-κB, Smad, and Nrf2 (Yuan et al., 2018; Vakili et al., 2020; Wong et al., 2020). Kaempferol, dietary bioflavinoid, existed in numerous types of plants. Kaempferol supplementation possesses the effect of regulating bone metabolism. Kaempferol exerts its bone protective effect by inhibiting adipogenesis, inflammation, oxidative stress, osteoclastic autophagy, and osteoblastic apoptosis via potential signaling pathways including BMP-2, NF-κB, MAPK, and mTOR signaling pathways (Kim et al., 2018; Liu et al., 2021; Wong et al., 2019). Beta-sitosterol existed in multiple traditional Chinese medicine herbs, involved in bone volume regulation by improving the expression of OPG and inhibiting the expression of RANKL (Ruangsuriya et al., 2020). On the other hand, beta-sitosterol was found to have an effect to accelerate the bone union of fracture in OVX rats, and this effect is highly possibly related to its affinity with estrogen and estrogen receptor (Chauhan et al., 2018). Pinoresinol has a similar chemical structure to estradiol. It was identified as promote proliferation and osteogenic markers via the cAMP signaling pathway *in vitro* (Jiang et al., 2019). Also, pinoresinol treatment up-regulated osteogenic differentiation and mineralization of the extracellular matrix via BMP-2/MAPK/β-catenin signaling pathway (Park et al., 2020). Medioresinol was not found to be effective in treating PMOP but was reported that possessed antifungal function, leishmaniasis activity, and cardiovascular disease risk reduction (Hwang et al., 2012; Hwang et al., 2013). All this literature supported the above-mentioned consequences of Bu-Yang herbs promoted BMD and bone microarchitecture in treating PMOP. However, the most basic and conservative mechanism shared by different Bu-Yang herbs in treating PMOP is still not elucidated.

Molecular docking analysis predicted the possible interaction between identical chemical compounds of BYD with ESR1. Network pharmacology predicted compounds shared by more than single herb in BYD, including quercetin, kaempferol, beta-sitosterol, pinoresinol, medioresinol. The compounds identified by LC-MS analysis of BYD, including icarrin, quercetin, pinosterol, and kaempferol. In short, icarrin, quercetin, kaempferol, beta-sitosterol, pinoresinol and medioresinol were used for molecular docking analysis to predict the possible interaction with ESR1. Detailed information was shown in Supplementary Figure S3B; Supplementary Table S4. As shown in Supplementary Table S4, results illustrated that following identical chemical compounds, including quercetin, kaempferol, beta-sitosterol, pinoresinol, medioresinol, had a potential of interaction with ESR1. Generally, with combination of results of LC-MS, showed in Supplementary Table S3, the existed compounds which has a potential interaction with ESR1 were quercetin, kaempferol and pinoresinol. These 3 compounds might be the main compounds of BYD in treatment of PMOP.

From the integrated drug target prediction and pathway analysis, ZJP may exert its antitumor effects on HCC via the regulation of cell proliferation and survival, which was characterized as the important mechanism of liver cancer progression (Guo et al., 2019). Signaling pathways that control multiple processes, such as cell proliferation, invasion, metastasis, and angiogenesis, are commonly dysregulated in the pathological progression of HCC, which have become an important source of targets from a therapeutic perspective in HCC treatment (Guo et al., 2019).

Integrated “herbs-compounds-targets” network (Supplementary Figure S2) and KEGG pathway analysis (Figure 2) predicted that BYD might possess its anti-osteoporosis effect on PMOP via the activation of ESR1 and estrogen signaling pathway. Estrogen deficiency was characterized as the pivotal mechanism of PMOP (Shen et al., 2020; Yang et al., 2020). Estrogen replacement therapy had viewed as an effective but unsafe therapy for its severe side effects of up-regulating the potential of cancer (Rachner et al., 2011). However, ESR1 and estrogen signaling pathway, as the main targets and mechanism of estrogen replacement therapy in treating PMOP, still attract much attention of researchers for which could be activated by natural products (Chiavarini et al., 2020; Zhou et al., 2021). As predicted in network pharmacology analysis, BYD may exert its anti-PMOP effect via ESR1 and estrogen signaling pathways. For further validation of the prediction, we investigated the curative effects of BYD on osteoblasts *in vitro* and on OVX-induced PMOP rats *in vivo*. The *in vivo* results demonstrated that BYD treatment significantly increased osteogenic markers including serum ALP, OPG, and PINP, improved BMD, and ameliorated bone microarchitecture damage (Figure 3). Additionally, the anti-PMOP effect of a high dosage of BYD is not worse than 487.5 μg/kg/d β-estradiol treatment. The *in vitro* results demonstrated that BYD treatment improved proliferation, osteogenic marker, mineralized nodules formation of osteoblasts (Figure 4). Furthermore, β-catenin and its downstream gene cyclinD1 were both up-regulated *in vitro* and *in vivo*, consistent with the up-regulation with ESR1. Interestingly, inhibition of ESR1 *in vitro* blocked the BYD-induced β-catenin activation and a series of osteogenic phenotypes *in vitro*.

PMOP is characterized by BMD loss, microarchitecture damaged, and increasing friability of bone (Xu et al., 2018). OVX-rats, as a PMOP animal model, is a well-accepted method (Chen et al., 2018; Ominsky et al., 2017; Zhang et al., 2018). OVX rats replicated the main characteristics of PMOP, including BMD loss and microarchitecture damaged, consistent with our micro-CT scanning and 3-dimensional remodeling results, as shown in Figures 4D,F–I. However, increasing friability of bone could not be replicated in OVX-induced PMOP rats for its absence of the Haversian canal. Considering that the increasing friability of bone is a pathological consequence of BMD loss and microarchitecture damaged, rather than the essential mechanism of PMOP. The observation of BMD and microarchitecture is enough to elucidate the changes after treatment. Therefore, it is adequate to use OVX-rats to replicate the PMOP model *in vivo* to validate the effects of BYD.

Osteogenesis biomarkers, including ALP, OPG, and RUNX2, were detected in the current study. ALP, a binding transporter distributed on cytomembrane, facilitates pre-osteoblast differentiated to mature osteoblasts and contributed to calcium deposition in the early stage of skeleton formation activity (Li et al., 2019b). RUNX2 is an important regulator of osteoblasts, for its regulating effect on stem cell differentiating fate to osteoblasts (Yin et al., 2018). RUNX2 is highly expressed on the nucleus of the cell, especially around emerging trabecular bone. Besides, RUNX2 participates in the regulation of osteocalcin, osteopontin, and collagens, which means RUNX2 is not only involved in the generation of osteoblasts but the function of osteoblasts. OPG, secreted by osteoblasts, binds RANKL in a competitive antagonism way, suppressed the differentiation and maturation of osteoclasts (Pietschmann et al., 2016). *In vivo* tests, by detecting serum ALP, OPG, and expression of RUNX2, revealed BYD promoted osteogenesis activity. Therefore, osteoblasts, the basic unit of osteogenesis activity and bone formation, was introduced as the observing object *in vitro* experiments. We confirmed that the BYD promoted the proliferation and osteogenesis function of osteoblasts *in vitro*.

The Wnt/β-catenin signaling pathway is one of the most important signaling pathways involved in bone metabolism. Briefly, once the Wnt/β-catenin signaling pathway was activated, the β-catenin destruction complex disintegrated resulted in β-catenin accumulated and translocated to the nucleus from the membrane to activate downstream targeting genes, including cyclinD1 and RUNX2. Immunofluorescence, Western blot, and RT-PCR were conducted to confirm the location and quantification of β-catenin. Results demonstrated BYD up-regulated expressions of β-catenin and promoted nucleus translocation of β-catenin, as well as the up-regulation of cyclinD1 and RUNX2 *in vitro* and Vivo. Inhibition of ESR1 by ICI182, 780 blocked the activation and up-regulation of β-catenin, cyclinD1 and RUNX2, suggested that BYD promotes bone formation by activating the β-catenin signaling pathway and its downstream targets, which depends on ESR1.

ESR1 is one of the two subtypes of estrogen receptor, which resident in bone and participates in bone metabolism (Krela-Kaźmierczak et al., 2019). ESR1 expression was found to be down-regulated in OVX-induced PMOP rats, this could be viewed as the result of OVX-induced estrogen deficiency. *In vivo*, BYD treatment significantly promoted ESR1 mRNA and protein expressions, just as β-estradiol treatment. *In vitro*, inhibition of ESR1 reversed the promotion of bone formation induced by BYD. Results validated the prediction of ESR1 being the main target gene of BYD in treating PMOP. Interestingly, the anti-PMOP effect of BYD was observed to be similar to β-estradiol, an artificial estrogen. Considering the ESR1 was predicted and validated to be the main target of BYD, we would like to infer that Bu-Yang herbs, represented by BYD, might possess natural phytoestrogens compounds. LC-MS analysis identified several representative chemical compounds resident in BYD, including icariin, pinoresinol, quercetin, and kaempferol. Previous studies illustrated the estrogen-like effects of extracts of certain natural products (Wang et al., 2018; Yuan et al., 2018; Sharma and Nam, 2019), this could be the possible reason that accounts for the anti-PMOP effects of BYD.

In the current study, literature reviewing revealed that Bu-Yang herbs were most frequently used in PMOP treatment among all TCM herbs, represented by YYH, DZ, BGZ, and TSZ, but the exact mechanisms were not elucidated. Network pharmacology analysis was conducted and found that the potential target, ESR1, might be the key help us understanding the mechanism of Bu-Yang herbs treating PMOP. Therefore, OVX rats were introduced as the PMOP animal model to validate the effects and mechanisms of BYD in treating PMOP. Results suggested that BYD treatment reversed the BMD loss and microarchitecture damaged induced by OVX in a dose-dependent manner. This effect is not worse than low dosage treatment of β-estradiol, a kind of estrogen. Moreover, BYD activated β-catenin signaling pathway to up-regulated RUNX2 expression via ESR1. This could be the possible mechanism of BYD in treating PMOP. *In vitro* study introduced ICI182, 780, an inhibitory of ESR1. The results demonstrated that BYD activated β-catenin signaling pathway and RUNX2 expressions via ESR1, resulted in promoting osteogenesis phenotype on osteoblasts. Inhibition of ESR1 blocked the trend, suggested that the osteogenic effects of BYD have mainly relied on ESR1. To sum up, Bu-Yang herbs protected bone mineral density and microarchitecture from estrogen deficiency via ESR1, the downstream mechanism might be related to activation of the β-catenin signaling pathway.

In conclusion, Bu-Yang herbs alleviated BMD loss and microarchitecture damaged in OVX-induced PMOP rats via ESR1. BYD possesses an estrogen-like effect, which up-regulates ESR1, activates β-catenin accumulation in osteoblasts, and promotes osteogenic activity. This natural estrogen-like effect of BYD was observed to be no worse than the low dosage of β-estradiol in treating PMOP rats. Further study should be done to look into the exact chemical compounds of BYD and the possibility of potential clinical use of BYD.

## Data Availability

The original contributions presented in the study are included in the article/Supplementary Material, further inquiries can be directed to the corresponding authors.
