# Supplementary material for: Integrated Strategy of Network Pharmacological Prediction and Experimental Validation Elucidate Possible Mechanism of Bu-Yang Herbs in Treating Postmenopausal Osteoporosis via ESR1
Source: Front Pharmacol. 2021 May 11;12:654714. doi: 10.3389/fphar.2021.654714 (PMC8144472; doi:10.3389/fphar.2021.654714)
Supplement: Supplementary file 1 [file DataSheet1.docx]

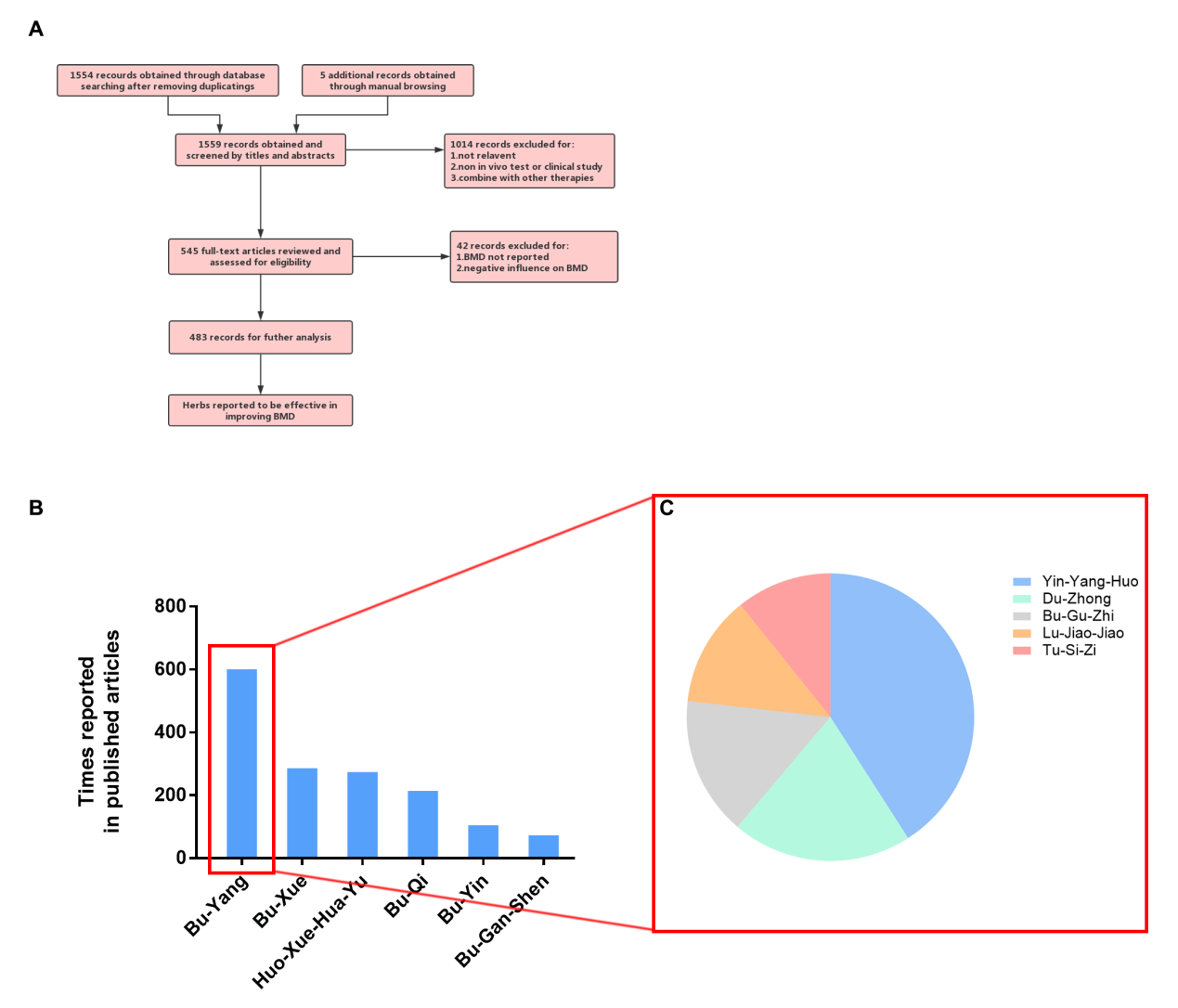


Supplementary Figure 1 Literature review showed that Bu-Yang herbs were most frequently used TCM herbs in PMOP treatment. (A) Literature review strategy. All records were reviewed by 2 independent reviewers. (B) Major categories of TCM herbs reported the in published articles regarding TCM treating PMOP. Bu-Yang: Tonifying Yang, Bu-Xue: Nourishing Blood, Huo-Xue-Hua-Yu: activating blood circulation to dissipate blood stasis; Bu-Qi: Tonifying Qi; Bu-Yin: Nourishing Yin. (B) Most frequently used Bu-Yang herbs. Yin-Yang-Huo: *Epimedium Brevicornum;* Du-Zhong: *Cortex Eucommiae;* Bu-Gu-Zhi: *Psoralea Corylifolia;* Tu-Si-Zi: *Semen Cuseutae.*


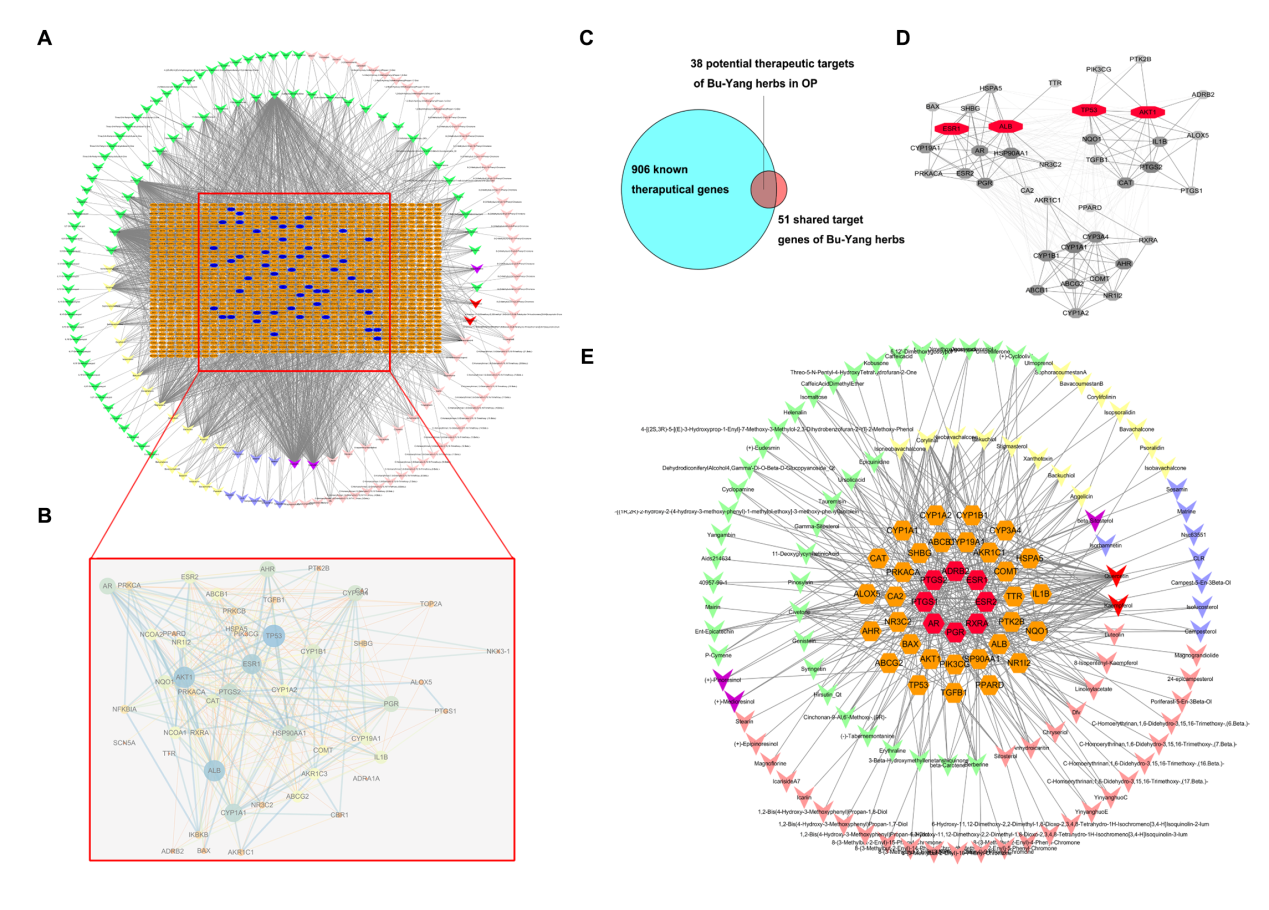


Supplementary Figure 2 Network pharmacology analysis of YYH, DZ, BGZ and TSZ. (A) Compound-Compond-targets network of YYH, DZ, BGZ and TSZ. Orang ellipse represents the putative targets of YYH, DZ, BGZ and TSZ, 51 blue ones represent the shared putative target genes of all 4 herbs. (B) Protein-Protein Interaction network of 51 shared target genes. (C) The venn analysis revealed 38 potential therapeutic targets between Bu-Yang herbs and PMOP. (D) ESR1, ALB, TP53 and AKT1 were hub genes in the PPI network of 38 therapeutic target genes. (E) Bu-Yang herbs-known postmenopausal osteoporosis targets visualized network. V represents the chemical compounds of YYH, DZ, BGZ and TSZ, the pink V represents the chemical compounds of YYH, the green V of DZ, the yellow V of BGZ and the light purple V of TSZ. The Vs line in the inner circle represent the chemical compounds interact with more putative target genes compared to the ones in the outer circle. Besides, the red V represents the chemical compounds shared by 2 different herbs, the dark purple V for 3 different herbs. The hexagon represents the 38 cross-talking genes and red ones stand for more interactions with chemical compounds of Bu-Yang herbs.


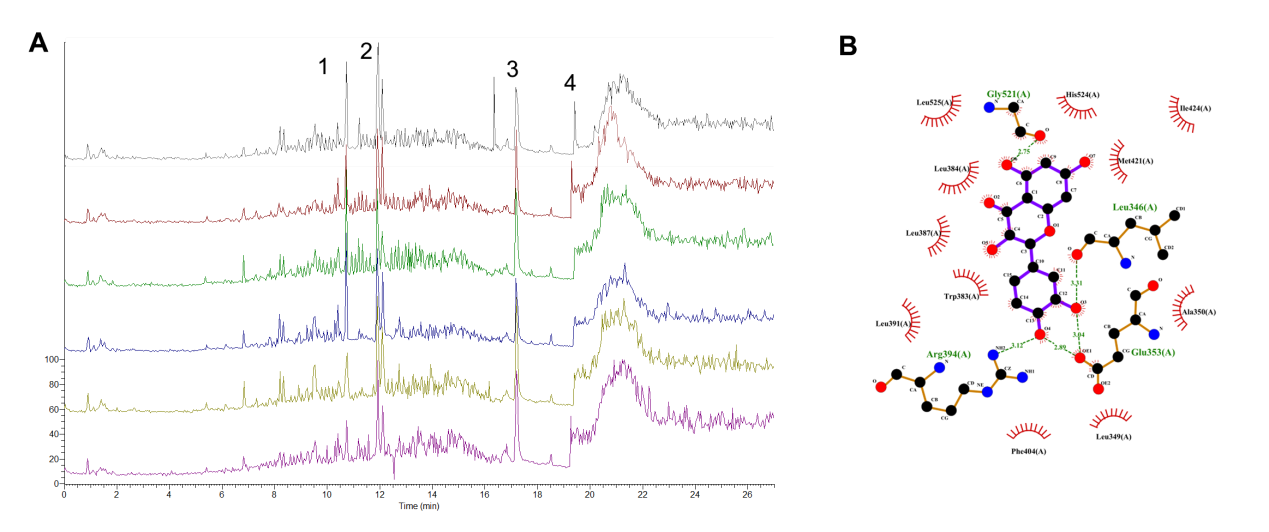


Supplementary Figure 3 (A) LC-MS analysis results of BYD. 1: icariin, 2:pinoresinol, 3:quercetin, 4:kaempferol. Gradient elution program was (time/B%): 0-1 min, 2%; 1-5 min, 2%-20%; 5-10 min, 20–50%; 10-15 min, 50-80%; 15–20 min, 80–95%; 20-25 min, 95%; 26–30 min, 2%. (B) Molecular docking prediction of chemeical compounds contained by at least two kinds of Bu-Yang herbs ((+)-Pinoresinol, beta-sitosterol, kaempferol, medioresinol and quercetin) with ESR1.


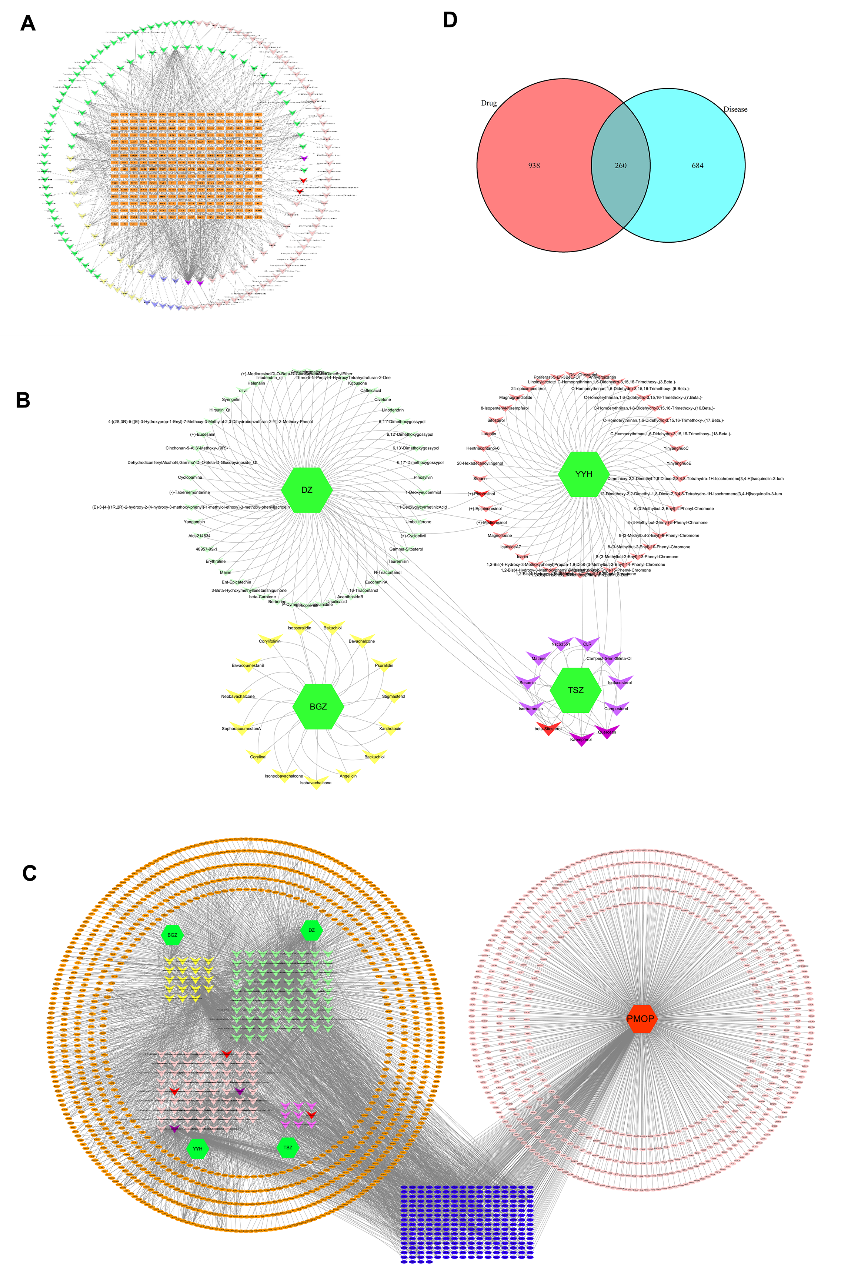


Supplementary Figure 4 (A) Chemical compounds-potential targets (genes which were both predicted to be the potential targets and also the known related genes of PMOP) network. (B) Herb-compounds network. (C) Herb-compounds-target-disease network. (D) Venn analysis of BYD and PMOP. V represents the chemical compounds of YYH, DZ, BGZ and TSZ, the pink V represents the chemical compounds of YYH, the green V of DZ, the yellow V of BGZ and the light purple V of TSZ. The Vs line in the inner circle represent the chemical compounds interact with more putative target genes compared to the ones in the outer circle. Besides, the red V represents the chemical compounds shared by 2 different herbs, the dark purple V for 3 different herbs. The hexagon represents the 38 cross-talking genes and red ones stand for more interactions with chemical compounds of Bu-Yang herbs. The green hexagon represents the herb of BYD. The orange V ellipse represents the targets of the BYD. The pink ellipse represents the known related targets of PMOP. The red hexagon represents the PMOP disease.

Supplementary Table 1 Prime Sequences

| Gene | Forward | Reverse |
| --- | --- | --- |
| Runx2 | 5'- CGGTGCAAACTTTCTCCAGG -3' | 5'-TTGCAGCCTTAAATGACTCGG -3' |
| β-catenin | 5'-GCGACTAAGCAGGAAGGGAT-3' | 5'-CCCACTTGGCACACCATCAT-3' |
| CyclinD1 | 5'-TCAAGTGTGACCCGGACTG-3' | 5'GACCAGCTTCTTCCTCCACTT-3' |
| ESR1 | 5'- TCTGCCTTGATCACACACCG -3' | 5'- GGATGAGCCACCCTGCTG -3' |

Supplementary Table 2 Putative target genes possessed high interactivity with chemical compounds.

| Target genes | Interacting relationship with chemical compounds |
| --- | --- |
| PTGS2 | 41 |
| ESR1 | 36 |
| AR | 34 |
| PTGS1 | 27 |
| PGR | 27 |
| SCN5A | 23 |
| CALM3 | 21 |
| NCOA2 | 19 |
| ADRB2 | 18 |
| KCNH2 | 18 |
| ESR2 | 17 |
| PRSS1 | 15 |
| NOS2 | 14 |
| ACHE | 14 |
| RXRA | 14 |
| DRD2 | 13 |
| NR3C2 | 12 |
| TOP2A | 11 |
| ADRA2A | 11 |
| F7 | 11 |

Supplementary Table 3 Representative chemical compounds of Bu-Yang decoction

| NO. | Compounds | t_R_/min | m/z | Chemical formula | Content(ug/ml) |
| --- | --- | --- | --- | --- | --- |
| 1 | Icariin | 10.73 | 363.06937 | C_33_H_40_O_15_ | 32.431 |
| 2 | Quercetin | 11.91 | 151.00115 | C_15_H_10_O_7_ | 227.45 |
| 3 | Pinoresinol | 17.34 | 137.05823 | C_20_H_22_O_6_ | 141.532 |
| 4 | Kaempferol | 19.01 | 153.0169 | C_15_H_10_O_6_ | 82.758 |

Supplementary Table 4 Molecular docking prediction

| NO. | Chemical compounds | Score |
| --- | --- | --- |
| 1 | (+)-Pinoresinol | -7.5 |
| 2 | beta-sitosterol | -6.8 |
| 3 | Kaempferol | -8.4 |
| 4 | medioresinol | -5.0 |
| 5 | Quercetin | -8.5 |
| 6 | Icarrin | -3.5 |
